# Monensin causes dose dependent inhibition of *Mycobacterium avium *subspecies *paratuberculosis *in radiometric culture

**DOI:** 10.1186/1757-4749-1-4

**Published:** 2009-02-09

**Authors:** Robert J Greenstein, Liya Su, Robert H Whitlock, Sheldon T Brown

**Affiliations:** 1 Laboratory of Molecular Surgical Research, VAMC Bronx, NY (112), 130 West Kingsbridge Road, Bronx, NY 10468, USA; 2 VAMC Bronx NY, 130 West Kingsbridge Road, Bronx, NY 10468, USA; 3 School of Veterinary Medicine, University of Pennsylvania, 382 West Street Road, Kennett Square, PA 19348, USA

## Abstract

**Background:**

*Mycobacterium avium *subspecies *paratuberculosis *(MAP) causes a chronic wasting diarrheal disease in ruminants called Johne's disease, that is evocative of human inflammatory bowel disease (IBD). Agents used to treat IBD, called "anti-inflammatories", immuno-modulators" and "immuno-suppressants" inhibit MAP growth in culture. We concluded that, unknowingly, the medical profession has been treating MAP since sulfasalazine's introduction in 1942. Monensin, called a "Growth Enhancer" in cattle, ameliorates Johne's disease without a documented mechanism of action. We hypothesized that Monensin would inhibit MAP in culture.

**Methods:**

Using the radiometric ^14^CO_2 _Bactec^® ^system, that expresses mycobacterial growth in arbitrary growth index (GI) units, we studied the effect of Monensin on the growth kinetic of MAP isolated from humans with IBD ("Dominic", "Ben" & UCF-4) and cattle with Johne's disease (303 & ATCC 19698.) Results are expressed as percent inhibition of cumulative GI (%–ΔcGI).

**Results:**

The positive control Clofazimine inhibits every strain tested. The negative controls Cycloheximide & Phthalimide, have no inhibition on any MAP strain. Monensin has dose dependent inhibition on every MAP strain tested. The most susceptible human isolate was UCF-4 (73% – ΔcGI at 1 μg/ml) and bovine isolate was 303 (73% – ΔcGI at 4 μg/ml.) Monensin additionally inhibits *M. avium *ATCC 25291 (87% – ΔcGI at 64 μg/ml) & BCG (92% – ΔcGI at 16 μg/ml).

**Discussion:**

We show that in radiometric culture the "Growth Enhancer" Monensin causes dose dependent inhibition of mycobacteria including MAP. We posit that the "Growth Enhancer" effect of Monensin may, at least in part, be due to inhibition of MAP in clinical or sub-clinical Johne's disease.

## Background

In ruminants worldwide *Mycobacterium avium *subspecies *paratuberculosis *(MAP) causes Johne's disease [1], which is evocative of inflammatory bowel disease (IBD) in humans [2]. As of 2007, 68% of all US cow herds had at least one environmental sample that cultured positive for MAP, rising to 95% in herds of > 500 cows [3]. The financial cost of Johne's disease to the agricultural industry, in the USA alone, is estimated to be > $200 million a year.

Humans are continually exposed to viable MAP, as MAP has been cultured from commercially available pasteurized milk in the US [4], and Europe [5] and is found in potable chlorinated municipal water in the US [6] and Europe [7]. Although controversial, there is mounting concern [8] and increasingly compelling data that MAP may be zoonotic. [9,10] Recently, the agents 5-ASA [11], azathioprine [12], 6-MP [12,13], methotrexate [13], cyclosporine A, [14] Rapamycin [14] and Tacrolimus [14], that are referred to as "anti-inflammatory" "immuno-modulator" and "immunosuppressants" by physicians, have been shown to cause dose dependent inhibition of MAP in culture. Corroborative evidence that MAP may be zoonotic are that, in humans, the most potent "antiMAP" agents in culture [12-14], actually clear MAP DNA from the blood of individuals with Inflammatory Bowel Disease [15]. We suggested [11,13,14] that, unknowingly, the medical profession had been treating MAP since 1942, when Nana Svartz introduced sulfasalazine into clinical practice [16].

Introduced in 1967, Monensin [17] is acknowledged as an anti-coccidicidal agent for poultry [18-20]. Approved as a "Growth Enhancer" antibiotic in the USA, the UK and Europe [21], Monensin accounts for 13% of the total subtherapeutic livestock antibiotic usage in the USA [22]. Eukaryotic metabolic effects of Monensin, include inhibition of endosome acidification [23] Na+ ionophore enhancement [24] including action on *Leishmania donovani*. [25] and possibly prokaryotes [23,26]. In ruminants the administration of Monensin results in improved energy balance [27], decreases methanogenesis [28], increases plasma urea-N [29], and increased milk production in lactating cows [30]. It is of considerable interest that the use of Monensin results in amelioration of pathology [21,31-33] and results in clinical improvement in animals with Johne's disease [34,35].

We hypothesized that Monensin, in addition to its protean anti-coccycidal [18-20] and eukaryotic effects [23-25,36] may additionally inhibit prokaryotes [26], in particular MAP. If correct, ruminant "Growth Enhancement" may in part be consequent to "antiMAP" antibiotic activity in cattle with clinical or sub-clinical Johne's disease. To test this hypothesis, we studied the effect of Monensin on mycobacteria including *M. avium *and its subspecies *paratuberculosis*, using our previously validated culture inhibition methods [11,13,14].

## Methods

This study was conducted as an approved protocol of the Research and Development Committee of the VAMC Bronx approved protocol (0720-06-038.) Inhibition studies were performed on eight mycobacterial strains in culture, as reported. [11,13,14] Five strains were MAP, of which three were isolated from humans with IBD, "Dominic" (ATCC 43545), Ben, (ATCC 43544) [37] & UCF-4 (gift of S. Naser Orlando FL.) Two were MAP bovine isolates, ATCC 19698 (ATCC Rockville MD) & 303 (gift of M. Collins. Madison WI) [38]. The *M. avium *subspecies *avium *strains (subsequently referred to as *M. avium*) were ATCC 28291 (ATCC) and *M. avium *101 [39]. To study the effect on the *M. tuberculosis *complex we used a Biosafety level II surrogate, BCG *M. bovis *Karlson & Lessel (ATCC 19015.) Agents (all from Sigma, St Louis MO) were dissolved in DMSO [11,13] with a final concentration in every Bactec vial, irrespective of the amount of agent in a vial, of 3.2% DMSO.

Quantifying mycobacterial growth and the effect of agents tested, using the radiometric ^14^CO_2 _Bactec 460 system, has previously been reported in detail [11,13,14]. In brief, the daily Growth Index (GI) for each vial is obtained until any vial reaches the instrument maximum of GI of "999." Daily GI's are summated until the day prior to any vial reaching "999." The effect (or lack thereof) of each agent is presented as the percent decrease in cumulative Growth Index (cGI) units (% – ΔcGI) ± SD [11] (when necessary, see individual figures).

## Results

**Figure 1:**
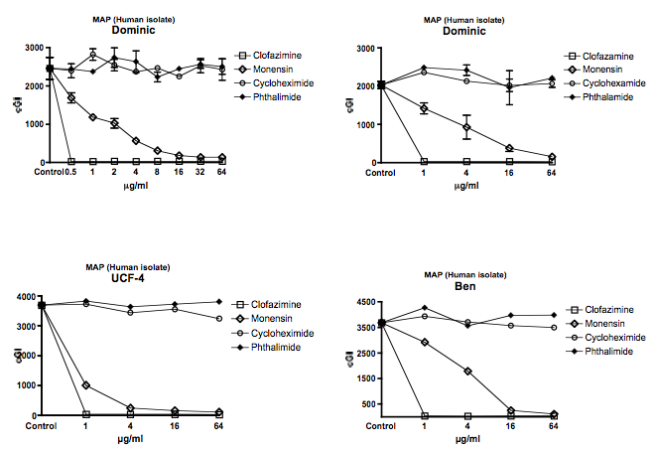
**Presents data for the three human isolates of MAP from patients with Crohn's disease**. All three have dose dependent inhibition, albeit to varying degrees for Dominic, UCF-4 and Ben. Error bars are ± SD.

**Figure 2:**
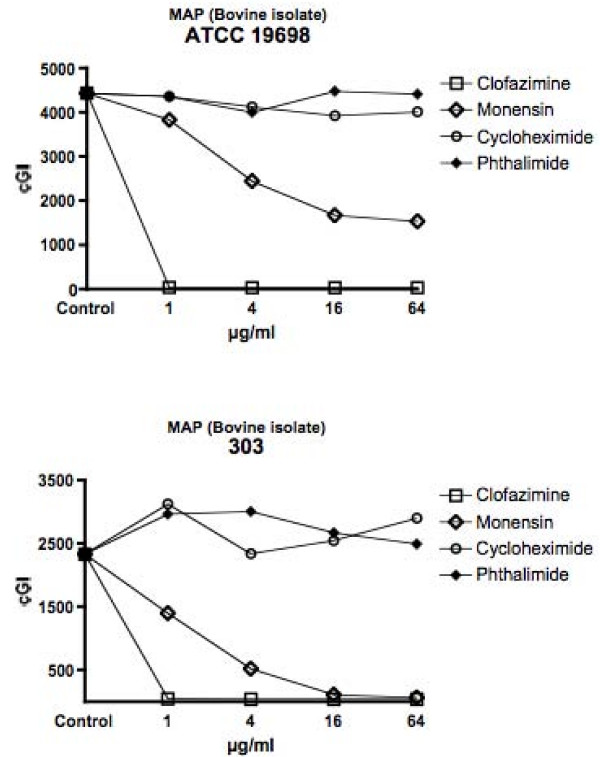
**Presents the dose dependent inhibition data for Monensin on isolates of MAP from cows with Johne's disease, ATCC 19698 and 303**. Error bars are ± SD.

**Figure 3:**
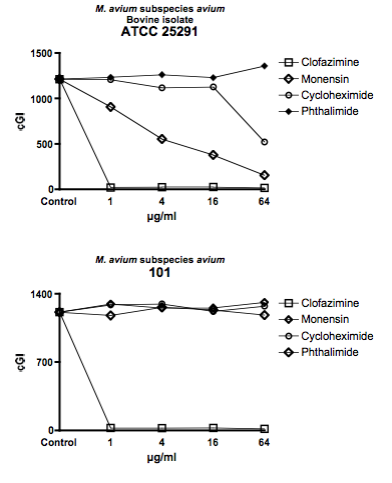
**Shown are the data from two Control strains, *M. avium *ATCC 25291 (Bovine source) and 101 **[39]. Note that Monensin, uniquely in this study has no dose dependent inhibition on *M. avium *subspecies *avium *101.

**Figure 4:**
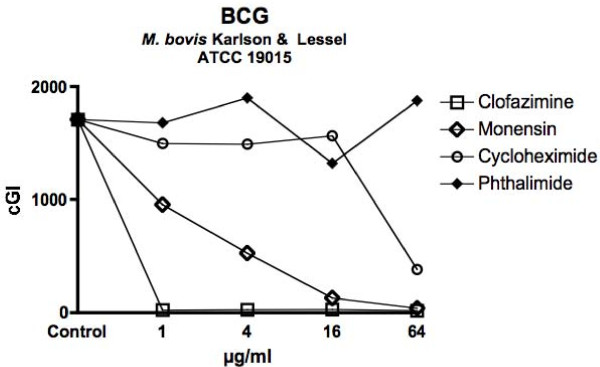
**With BCG, our Biosafety level II surrogate for the *M. tuberculosis *complex, Monensin exhibits doses dependent inhibition**.

For ease of comprehension, data are presented in two ways: For individual mycobacterial strains, data are presented as Figures using the cumulative Growth Index (cGI). For individual chemicals the same data, recalculated as %–ΔcGI [11,13], are presented as Tables. Figure 1 = MAP isolated from humans with Crohn's Disease. Figure 2 = Bovine Isolates of MAP from animals with Johne's disease. Figure 3 is *M. avium *subspecies *avium*. Figure 4 presents data for BCG. Table 1 is the positive antibiotic control Clofazimine (used in leprosy [40] and clinical trials of Crohn's disease [41,42].) The negative controls are the gluterimide antibiotics cycloheximide (Table 2) and phthalimide (Table 3.) The study results on Monensin are presented in Table 4.

**Table 1:** Positive control

Clofazimine								
%–ΔcGI								
Clofazimine	MAP					M. avium		M. bovis

μg/ml	Human			Bovine		Bovine		BCG

	Dominic	UCF-4	Ben	19698	303	25291	101	19015

1	-99%	-99%	-99%	-99%	-99%	-98%	-98%	-99%

4	-99%	-99%	-99%	-99%	-99%	-98%	-98%	-98%

16	-99%	-99%	-99%	-99%	-99%	-98%	-98%	-98%

64	-99%	-99%	-99%	-99%	-99%	-99%	-99%	-99%

Data are presented for a single agent, Clofazimine, the positive antibiotic control. Data are given for a representative experiment of each mycobacterial strain studied. Note maximal inhibition at 1 μg/ml. for every strain. %–ΔcGI = Percent decrease in cumulative Growth Index compared to solvent control (see Methods.)

**Table 2:** Negative Control

Cycloheximide								
Cycloheximide	MAP					M. avium		M. bovis

μg/ml	Human			Bovine		Bovine		BCG

	Dominic	UCF-4	Ben	19698	303	25291	101	19015

1	15%	1%	7%	-2%	3%	0%	6%	-12%

4	-4%	-7%	1%	-7%	4%	-8%	7%	-13%

16	-8%	-4%	-3%	-12%	-5%	-7%	1%	-8%

64	-1%	-12%	-5%	-9%	-4%	-57%	5%	-78%

Data are presented for a single agent, the gluterimide antibiotic Cycloheximide, a negative antibiotic control. Data are given for a representative experiment of each mycobacterial strain studied. Note that there is no strain exhibiting dose dependent inhibition within the range tested. %–ΔcGI = Percent decrease in cumulative Growth Index compared to solvent control (see Methods.)

**Table 3:** Negative Control

Phthalimide								
%–ΔcGI								
Phthalimide	MAP					M. avium		M. bovis

μg/ml	Human			Bovine		Bovine		BCG

	Dominic	UCF-4	Ben	19698	303	25291	101	19015

1	-1%	4%	16%	-2%	-2%	2%	-3%	-2%

4	0%	-2%	-4%	-10%	3%	4%	4%	11%

16	1%	1%	8%	1%	-13%	1%	2%	-23%

64	4%	3%	8%	0%	6%	12%	-2%	10%

Data are presented for a single agent, Phthalimide another negative antibiotic control. Data are given for a representative experiment of each mycobacterial strain studied. Note that there is no strain exhibiting dose dependent inhibition within the range tested. %–ΔcGI = Percent decrease in cumulative Growth Index compared to solvent control (see Methods.)

**Table 4:** The study results on Monensin

Monensin								
%–ΔcGI								
Monensin	MAP					M. avium		M. bovis

μg/ml	Human			Bovine		Bovine		BCG

	Dominic	UCF-4	Ben	19698	303	25291	101	19015

1	-52%	-73%	-21%	-14%	-21%	-25%	7%	-44%

4	-77%	-93%	-51%	-45%	-73%	-54%	3%	-69%

16	-93%	-96%	-93%	-62%	-94%	-69%	4%	-92%

64	-94%	-97%	-97%	-65%	-97%	-87%	8%	-98%

Data are presented for the agent evaluated in this study, the "Growth Enhancer" Monensin. Data are given for a representative experiment of each mycobacterial strain studied. Note the dose dependent inhibition in every mycobacterial strain except *M. avium *subspecies *avium *101. The dose dependent inhibition was observed in each of the 21 experiments where MAP was the strain studied (replicative data not presented.) %–ΔcGI = Percent decrease in cumulative Growth Index compared to solvent control (see Methods.)

In total we performed 29 culture inhibition experiments that included Monensin, of which 21 involved MAP that had been isolated from either ruminants or humans. In every case where Monensin was studied for its effect on MAP, dose dependent inhibition was observed. For brevity and clarity, representative studies are presented. The positive control, clofazimine causes dose dependent inhibition; > 98% – ΔcGI in every strain tested by 1 μg/ml (Figures 1, 2, 3, 4 & Table 1.) A negative control Cycloheximide has no inhibition on any MAP strain at the doses used in these studies (Figures 1 &2 & Table 2.) In contrast, when tested against *M. avium *ATCC 25291, Cycloheximide has no effect between 1 & 16 μg/ml, but does have 57% – ΔcGI at 64 μg/ml (Figures 3 & Table 2.) Similarly, against BCG, Cycloheximide has no dose dependent inhibition between 1 & 16 μg/ml, but does have 78% – ΔcGI at 64 μg/ml (Figures 4 & Table 2.) Phthalimide, the other negative control, has no dose dependent inhibition against any of the eight mycobacterial strains evaluated (Figures 1, 2, 3, 4 & Table 3.)

Monensin exhibits dose dependent inhibition on MAP in culture, whether isolated from humans with Crohn's disease (Figure 1 & Table 4) or cows with Johne's disease (Figure 2 & Table 4.) Of the human MAP isolates, UCF-4 was most susceptible, 73% – ΔcGI at 1 μg/ml (Figure 1 & Table 4) and Ben least inhibited (51% – ΔcGI at 4 μg/ml: Figure 1 & Table 4.) Of the two bovine MAP isolates, ATCC 19698 (45% – ΔcGI at 4 μg/ml) was less inhibited than 303 (73% – ΔcGI at 4 μg/ml: Figure 2 & Table 4.) Monensin additionally causes dose dependent inhibition on two of the three control mycobacterial strains; ATCC 25291 & BCG (Figures 3 &4 & Table 4.) The single exception to inhibition by Monensin is with the control *M. avium *subspecies *avium *strain 101 (Figure 3 & Table 3.)

## Discussion

The "anti-inflammatories" "immune-modulators' and "immune-suppressants" 5-ASA [11], azathioprine [12], 6-MP [12,13], methotrexate [13], cyclosporine A, [14] Rapamycin [14] and Tacrolimus [14], all cause dose dependent inhibition of MAP in culture. We suggest that, unlike the majority of antibiotics, which effect only prokaryotes, these agents inhibit both pro and eukaryotes. It is therefore possible that these terms "anti-inflammatories" "immune-modulators' and "immune-suppressants" are actually misnomers. We suggest that these appellations merely report normal secondary eukaryotic physiological effects, consequent to, unknowingly, treating an underlying prokaryotic infection. Specifically we suggest that since 1942, the medical profession has unknowingly been treating MAP infections when using these medications.

Our present data corroborate the culture finding of Brumbaugh *et. al*. [33]. With a single strain of MAP, in one culture experiment, there was no visually detectable growth at 30 days following inoculation. This was interpreted as showing a Monensin Minimum Inhibitory Concentration of 0.39 μg/ml against MAP. In our study, a distillation of 29 different experiments involving Monensin, of which 21 were conducted against five strains of MAP, we show that, in radiometric culture, Monensin causes dose dependent inhibition of MAP, one of two strains of *M. avium *subspecies *avium *and a single strain of *M. Bovis*.

The fact that MAP is the etiological agent in Johne's disease is uncontested. There have been a plethora of observation studies on the "metabolic" effects [26,28-30] when Monensin is used in cattle. Monensin is called a "Growth Enhancer" by both veterinarians as well as governmental agencies [21]. These culture inhibition studies offer a rational explanation for the healing of Johne's lesions [31,32] in ruminants with Johne's disease treated with Monensin. As mean prevalence of MAP in US dairy herds steadily increases, currently 68%, [3] it is probable that some of these observations were, unknowingly, made on animals with sub-clinical Johne's disease. We posit that at least some of the "Growth Enhancement" as well as metabolic effects that attends the use of Monensin in ruminants may be the physiological consequence of treating overt or covert MAP infections.

A plausible question is whether the appropriate use of antiMAP agents "cures" Johne's disease? The dose dependent inhibition of Monensin that we demonstrate, are compatible with Monensin being a bacteriostatic, rather than bactericidal antiMAP agent. Our data are therefore compatible with the observations that the use of Monensin ameliorates [32], but does not eradicate or clear MAP from animals with Johne's disease [31,33]. This would render Monensin analogous to another "antiMAP" agent, 5-ASA where the effect is demonstrably bacteriostatic [11]. In our culture system Monensin is not nearly as inhibitory as methotrexate and 6-MP [12,13], which clear MAP DNA from the blood of individuals with Inflammatory Bowel Disease [15]. We conclude that Monensin cannot be considered as a "cure" for a ruminant MAP infection.

This study does not address the inhibitory mechanism of Monensin on MAP. One possibility is that it is consequent to cell wall destruction, as occurs with coccidia [18]. Alternatively Monensin's antiMAP activity may be due to perturbation of obligate intracellular metabolic pathways such a DNA synthesis, as occurs with Methotrexate and 6-MP. Distinguishing between the two modes of action is important. In man, MAP exists in the cell wall deficient form (see [10] for review). In contrast, in ruminants it is the cell wall containing form of MAP that is readily identified [1]. We conclude that our present observations do not justify initiating human clinical trials of Monensin in putative zoonotic MAP infections such as Crohn's disease or ulcerative colitis. Such appropriately planned and authorized human trials should only be performed if the antiMAP action of Monensin is unequivocally shown to affect intracellular metabolic pathways of MAP and not its cell wall.

## Abbreviations

MAP: Mycobacterium avium subspecies paratuberculosis; IBD: inflammatory bowel disease; GI: Growth Index; cGI: cumulative Growth Index; % – ΔcGI: percent decrease in cGI units compared to control growth.

## Competing interests

RJG submitted provisional patents based on the hypotheses tested in prior studies. There is no conflict of interest with these Monensin data. STB was a member of the panel of the National Academy of Sciences of the USA that issued the report entitled "The Diagnosis and Control of Johne's disease (ISBN 0-309-08611-6). LS and RHW have no competing interests.

## Authors' contributions

RHW and RJG conceived the experiments. RJG and STB designed the experiments. LS and RJG performed the experiments. RJG, LS, RHW and STB analysed the data. STB and RJG contributed reagents/materials analysis equipment. RJG wrote the manuscript. All authors read and approved the final manuscript.
